# Effect of Lung Compliance-Based Optimum Pressure Versus Fixed Positive End-Expiratory Pressure on Lung Atelectasis Assessed by Modified Lung Ultrasound Score in Laparoscopic Gynecological Surgery: A Prospective Randomized Controlled Trial

**DOI:** 10.7759/cureus.40278

**Published:** 2023-06-12

**Authors:** Deeparaj L, Rakesh Kumar, Nishant Patel, Arshad Ayub, Vimi Rewari, Rajeshwari Subramaniam, Kallol Kumar Roy

**Affiliations:** 1 Anesthesiology, Pain Medicine and Critical Care, All India Institute of Medical Sciences, New Delhi, IND; 2 Obstetrics and Gynecology, All India Institute of Medical Sciences, New Delhi, IND

**Keywords:** postoperative pulmonary complication, lung ultrasound score, lung atelectasis, optimum peep, laparoscopic gynecological surgery

## Abstract

Background: Lung protective ventilation during the intraoperative period is now well established. However, the additional role of positive end-expiratory pressure (PEEP) during the intraoperative period remains uncertain in major laparoscopic gynecological surgery. The authors hypothesized that compliance-based optimum PEEP titration reduces postoperative lung atelectasis and improves outcomes.

Methods: Patients undergoing major laparoscopic pelvic gynecological surgeries with healthy lungs were randomized to the fixed PEEP group (PEEP 5 cm H_2_O and recruitment maneuver {RM}) and optimum PEEP group (compliance-based PEEP and RM). Lung ultrasound and arterial blood gas analysis were performed at four time points. Modified lung ultrasound scoring was done, and the same was used as means of assessing lung aeration and the amount of lung atelectasis. Postoperative supplemental oxygen requirement and duration were also assessed and compared.

Results: Lung ultrasound score (LUS) 30 minutes after extubation in fixed (Group F) and optimum (Group O) PEEP groups were median (interquartile range {IQR}) 3 (2-3) versus 1 (1-2), p=0.0001. Ventilatory parameters between Group F and Group O after lung recruitment were tidal volume (mean 357 mL {SD: 35} versus 362 mL {SD: 22}, p=0.46), PEEP (median, 5 cm H_2_O {IQR: 5-5} versus median 16 cm H_2_O {IQR: 14-18}), peak airway pressure (median 26 cm H_2_O {IQR: 24-28} versus median 30 cm H_2_O {IQR: 28-32} p<0.0001), plateau pressure (median 22 cm H_2_O {IQR: 20-24} versus median 26 cm H_2_O {IQR: 24-28} p<0.0001), static compliance (32.07±8.36 mL cm H_2_O-1 versus 39.58±8.99 mL cm H_2_O-1, p=0.0002). The number of patients requiring postoperative oxygen therapy to maintain SpO_2 _>94% after extubation in postanesthesia care unit (PACU) was statistically significantly greater in group F (39/41 {95%} versus 30/41 {73%}, p=0.007). Median (IQR) duration of oxygenation therapy in the first 24 hours of the postoperative period between Group F and Group O differed with statistical significance, with the median (IQR) values being 25 (20-30) minutes versus 10 (0-15) minutes (p<0.0001).

Conclusions: The modified lung ultrasound score significantly differed intraoperatively between the two groups, with lower scores in the optimum PEEP group. This has reflected improved postoperative outcomes in optimum PEEP group patients, with fewer patients requiring postoperative oxygen supplementation and reduced supplemental oxygen requirement duration.

## Introduction

Many major gynecological surgeries are routinely performed laparoscopically due to early recovery and fewer postoperative complications. However, respiratory mechanics have a significant compromise due to general anesthesia, pneumoperitoneum, and Trendelenburg position during the intraoperative period. These compromised respiratory mechanics may lead to postoperative pulmonary complications (POPC) and are associated with increased duration of hospital stay, morbidity, and mortality. POPC terms include a spectrum of respiratory complications encompassing lung atelectasis, consolidation, respiratory failure, aspiration pneumonitis, and acute respiratory distress syndrome (ARDS) [1]. Depending on how POPC is defined, the incidence of POPCs ranges from 6% to 80% following the emergence of general anesthesia [2].

Risk factors for developing POPC include duration of anesthesia, specific positionings such as Trendelenburg position in pelvic and robotic surgeries, high intraoperative FiO_2_, obesity, and inappropriate intraoperative mechanical ventilation [2]. General anesthesia itself reduces functional residual capacity and alters respiratory drive and muscle function [3]. Even in an otherwise uneventful anesthesia, 10-15% of lung tissue can develop collapse, especially in the dependent lung areas [4]. General anesthesia also causes a decrease in end-expiratory lung volume and diminished arterial oxygenation.

Laparoscopic gynecological surgeries are performed in the Trendelenburg position [5,6]. The physiological effect of Trendelenburg position, pneumoperitoneum, and general anesthesia adversely affect respiratory mechanics and cause the collapse of the dependent lung and worsening gas exchange [7-10].

In laparoscopic surgery, different ventilatory strategies have been studied to improve lung mechanics and oxygenation in recent years. Many have demonstrated low tidal volume and an external positive end-expiratory pressure (PEEP) to prevent tidal recruitment - derecruitment of alveoli leading to atelectotrauma [8]. A recent trial in morbidly obese patients demonstrated that the beach chair position and PEEP improved oxygenation during pneumoperitoneum creation [11]. Low tidal volumes reduce pulmonary stress and strain, whereas external PEEP and recruitment maneuver (RM) improved oxygenation by reopening collapsed segments and preventing atelectasis. The external PEEP application counterbalances the cranial diaphragm shift due to the pneumoperitoneum and Trendelenburg position, which increases functional residual capacity and improves lung compliance [12-14]. An optimum PEEP on a patient-to-patient basis is needed to avoid alveolar overdistension and associated hemodynamic instability.

So far, no study has been done on the effect of optimum PEEP based on lung compliance on lung atelectasis during general anesthesia in major laparoscopic gynecological surgeries. In this study, we tried to document the physiological effect of a ventilatory strategy used to counteract alveolar collapse induced due to pneumoperitoneum and Trendelenburg position. We hypothesized that with the use of optimum PEEP during the intraoperative period, there could be a significant decrease in lung atelectasis as assessed by the modified lung ultrasound score in the optimum PEEP group compared to the fixed PEEP group in laparoscopic gynecological surgery. The primary objective of this study was to assess the magnitude of postoperative (30 minutes after extubation) lung atelectasis by modified lung ultrasound score. The secondary objective was to investigate the incidence of intraoperative hypotension and bradycardia, driving pressure changes, and the number of patients and duration of supplemental O_2_ requirement in the postoperative period in postanesthesia care unit (PACU) and the first postoperative day. Additionally, lung ultrasounds were correlated to partial pressure of oxygen/fraction of inspired oxygen (P/F), BMI, alveolar-arterial gradient (A-a) gradient, and duration of pneumoperitoneum.

## Materials and methods

This is a single-center prospective randomized parallel-group trial conducted in a tertiary care institute of All India Institute of Medical Sciences, New Delhi. The protocol was registered in the National Clinical Trial Registry of India (www.ctri.nic.in; CTRI registration no. CTRI/2020/03/023896, reference no. REF/2019/12/029882), and permission was obtained from the Institute Ethics Committee before recruitment of the first patient. After obtaining informed written consent, 82 patients were randomized in a 1:1 ratio in two groups. Allocated treatment was given to all recruited patients, and complete outcome data were collected from all patients.

Adult female patients (aged between 18 and 65 years) of American Society of Anesthesiologists (ASA) physical status I or II, undergoing elective major laparoscopic gynecological surgery (defined by a duration of two or more hours) under general anesthesia in Trendelenburg position (200-300) were recruited in this study. Patients with BMI>30 kg/m^2^ or <18 kg/m^2^, known chronic obstructive pulmonary disease (COPD) or another chronic lung disease, known deformity of the chest wall or thoracic spine, patients with a history of previous thoracic surgery, patients with known pre-existing significant cardiac, renal and hepatic diseases were excluded from this study. Patients were randomized according to a computer-generated random number table in two groups, Group F and Group O. In Group F, fixed PEEP of 5 cm H_2_O was applied, whereas in Group O, optimum PEEP according to best static lung compliance was applied.

Allocations of the patients in the study group were kept inside a sealed opaque envelope. They were opened by a person not involved in this study after anesthesia induction and before the pneumoperitoneum's creation. Anesthesiologist, who was managing the patients, was not blinded to the group allocation. Investigators collecting the postoperative outcome data were blinded to the allocation.

All patients underwent a detailed pre-anesthetic examination per the institute protocol at least one day before surgery. Standard monitoring per the American Society of Anesthesiologists' recommendation (3-lead or 5-lead ECG per the patient's requirement, pulse oximetry, non-invasive blood pressure {NIBP}) was initiated in the operating room, and baseline vitals (heart rate, blood pressure, and SpO_2_) were recorded. Under local anesthesia, the radial artery was cannulated with a 20G catheter for intraoperative hemodynamic monitoring, and baseline arterial blood gas (ABG) analysis was done in room air.

General anesthesia was induced with intravenous fentanyl 2 mcg/kg and propofol 2-3 mg/kg, and tracheal intubation was facilitated by intravenous atracurium. Anesthesia was maintained with isoflurane in air-oxygen (FiO_2_=0.5), targeting an end-tidal concentration of 0.8-1.0 minimum alveolar concentration (MAC). Muscle relaxation was maintained with intermittent boluses of atracurium to maintain a train of four (TOF) count of 0-1. Multimodal analgesia was used for pain management as decided by the attending anesthesiologist. The attending anesthesiologist decided on the intraoperative fluid protocol. The total amount of intraoperative fluid (crystalloid, colloid, blood, and blood products) was recorded.

At the end of the surgery, the inhalation anesthetic was stopped, and the appropriate dosage of neostigmine and glycopyrrolate reversed the residual neuromuscular blockade. The trachea was extubated when patients were fully awake, and clinical end points of adequate reversal of neuromuscular blockade and a TOF ratio of 0.9 were reached. All patients received dexamethasone 8 mg and ondansetron 4 mg for postoperative nausea and vomiting (PONV) prophylaxis, except the diabetic patients who received only ondansetron.

Intraoperative ventilation strategy

After intubation, initially, all patients were ventilated with volume control ventilation (VCV) with a tidal volume of 6 mL/kg of ideal body weight (IBW) as per ARDS net formula, respiratory rate 12/minute, inspiratory:expiratory ratio 1:2, PEEP of 5 cm H_2_O, and inspiratory pause of 30% of the total inspiratory time. The respiratory rate was adjusted to maintain end-tidal CO_2_ between 35-40 mmHg. After final positioning (lithotomy and Trendelenburg of 20°-30°) and creation of pneumoperitoneum (intraabdominal pressure 12-14 cm H_2_O), lung recruitment was done in both groups according to the set protocol. After lung recruitment, PEEP was applied according to the allocated group.

Lung recruitment protocol

Lung recruitment was done in three steps. In the first step, after pneumoperitoneum, the ventilatory mode was changed from volume control ventilation (VCV) to pressure control ventilation (PCV) with pressure support of 20 cm H_2_O, a respiratory rate (RR) of 10 breaths per minute, and PEEP of 10 cm H_2_O. In the second step, PEEP was increased from 10 cm H_2_O to 15 cm H_2_O without a change in other parameters. In the third step, PEEP was increased from 15 cm H_2_O to 20 cm H_2_O without a change in other parameters. At each step, patients were ventilated for one minute. During the recruitment maneuver, continuous hemodynamic monitoring was done. Lung recruitment maneuver was discontinued if either one or more conditions were observed like a fall in SpO_2 _to 88% or an increase in heart rate to more than 140 beats/min, or decreased heart rate to less than 50 beats/min, or a fall in mean arterial pressure to less than 60 mmHg or decreased by more than 20% from baseline; or development of cardiac arrhythmia.

**Figure 1:**
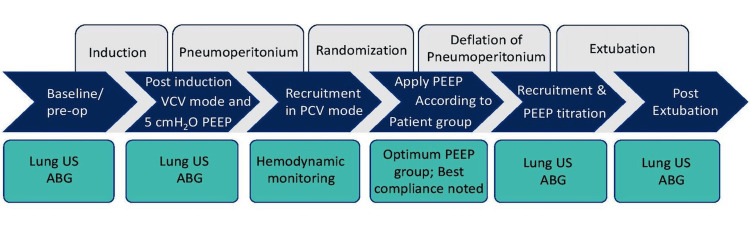
Positive end-expiratory pressure (PEEP) titration protocol. ABG: arterial blood gas

After lung recruitment, the ventilatory setting of patients was changed from PCV to VCV. In VCV, ventilatory setting was the same as before recruitment except for PEEP per group allocation. In Group F, PEEP of 5 cm H_2_O was applied throughout the surgery. In Group O, optimum PEEP was applied according to best static lung compliance. Best lung compliance was derived with the help of decremental PEEP titration. In this method, PEEP was decreased by 2 cm H_2_O at 45 seconds interval from 20 cm H_2_O until maximum lung compliance was achieved. An anesthesia machine calculated and displayed static lung compliance (Drager Primus; Lübeck, Germany: Drägerwerk AG & Co. KGaA). Peak airway pressure (PAP), plateau pressure (PP), driving pressure (DP), and lung compliance were noted after induction, before final positioning, after pneumoperitoneum but before lung recruitment, after lung recruitment, and after that, each 30-minute interval, after deflation of pneumoperitoneum and just before extubation. The lung recruitment maneuver was repeated every hour during pneumoperitoneum in both groups and PEEP was applied as per group. At the end of pneumoperitoneum, PEEP was again adjusted according to best lung compliance using the decremental PEEP titration method in Group O where a fixed PEEP of 5 cm H2O was applied in Group F. PEEP titration protocol is summarized in Figure 1.

In the case of intraoperative hypoxemia, optimum PEEP was planned to be used as a rescue strategy in fixed PEEP groups targeting SpO_2_>95%, and recruitment maneuver and increase in FiO_2_ were planned to be given in a stepwise manner in both groups. If hypoxemia persisted, a requirement of rescue therapy, if any, was supposed to be recorded.

Lung ultrasound score

**Figure 2:**
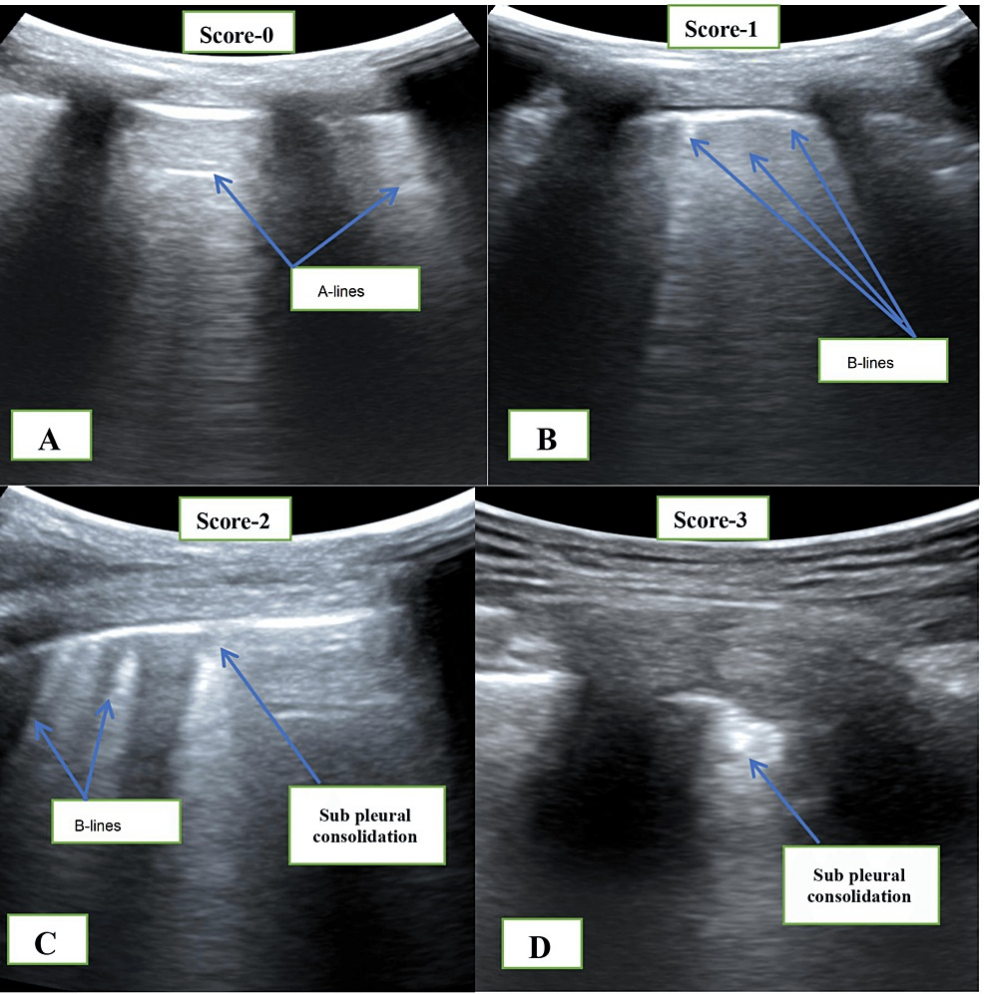
Modified lung ultrasound score based on the number of B-line and subpleural consolidation. (A) Score 0 - A-line and normal pleura; (B) score 1, 3 - B-line with a normal pleural line; (C) score 2, 3 - B-line and small subpleural consolidation; and (D) score 3 - large subpleural consolidation with significant air loss.

Lung ultrasound was performed by a single blinded investigator who had the experience of performing more than 50 perioperative lung ultrasounds under supervision before the initiation of this trial. Low-frequency curvilinear (2-5 MHz) or high-frequency, linear (6-13 MHz) probe of the SonoSite Fujifilm Ultrasound System (Bothell, WA: SonoSite, Inc.) was used for lung ultrasound scanning. As per the previous study, each side of the thorax was divided into six zones by two vertical (anterior and posterior axillary lines) and one imaginary horizontal line (passing above the nipple), so a total of 12 lung areas were scanned [1]. Intercostal spaces of each of these areas were scanned, and an image of the most pathologic area of each quadrant was saved to digital format for offline analysis and scoring. The scoring of lung ultrasound was done according to a modified lung ultrasound scoring (mLUS) system [1]. According to the mLUS system for A-profile or up to 2 B-line separated by regular pleural lines is scored zero. However, the score is one if there are three or more B-lines with or without small subpleural consolidation and a regular pleural line. The score is two if there are coalescent B-lines and or small subpleural consolidation with an irregular pleural line in between. The score is three; if extensive subpleural consolidation is more than 1x2 cm. Detailed ultrasound images were summarized in Figure 2. Based upon the mLUS scoring system, each area was scored from 0-3, so a minimum zero or maximum 36 score was possible. Lung ultrasound was performed at four predefined time points, such as before induction of general anesthesia (time point A), 10 minutes after induction of anesthesia but before positioning and creation of pneumoperitoneum (time point B), after deflation of pneumoperitoneum but before extubation (time point C), and in postanesthesia care unit (PACU) after 30 minutes of extubation (time point D).

Arterial blood gas analysis

Arterial blood gas analysis (ABG) was also done at points A through D, along with LUS, to ascertain the oxygenation status and alveolar-arterial gradient.

Postoperative management

After surgery, patients were managed in the PACU, and standard monitors were attached. For those patients who did not maintain oxygen saturation of more than 94%, supplemental oxygen through a face mask at 5 liters was given. Duration of supplementation of oxygen was recorded in both groups. Thirty minutes after the surgery, vital signs like heart rate (HR), NIBP, oxygen saturation (SpO_2_), and estimated delivered FiO_2_ were noted in the PACU. Lung ultrasound and ABG were also done 30 minutes after shifting to PACU. The severity of pain using an 11-point (0-10) numeric rating scale was also done. Patients were discharged from PACU after attaining Aldrete score ≥9 and shifted to the ward for postoperative management. Patients were monitored in the ward for the first postoperative day, and the duration of oxygen supplementation, if any, was recorded.

Sample size estimation

For sample size calculation 30% reduction in lung ultrasound score was considered clinically significant. At the end of the surgery, a lung ultrasound score was 8.52 (4.5) in PEEP of 5 cm H_2_O in one of the unpublished data from our institute. We assumed a power of 80% and an alfa of 0.05, a minimum of 37 patients in each group were required. If we assume there was an attrition of data of 10% in each group, a total of 41 patients were required in each group.

Statistical analysis of data

The distribution of data was tested with Kolmogorov-Smirnov analysis. Data were presented as mean and standard deviation (SD) and median and interquartile range for nominal and skewed data, respectively. Paired and unpaired t-tests were used to compare two unrelated nominal data, and Wilcoxon signed-rank test and Mann-Whitney U test were used to compare two unrelated skewed data. The difference in proportions was evaluated using Fisher's exact test, and a risk ratio with an associated 95% confidence interval (CI) was reported. Time-dependent respiratory variables and lung USG score were also modeled by a rank-based non-parametric method to identify the effect of the interaction of time and treatment allocation, and relative treatment effect (RTE) was reported. All analyses were conducted in R version 4.2.1 (R Development Core Team; R Foundation for Statistical Computing, Vienna, Austria) and STATA version 13 (College Station, TX: StataCorp LLC) for Mac OS.

## Results

**Figure 3:**
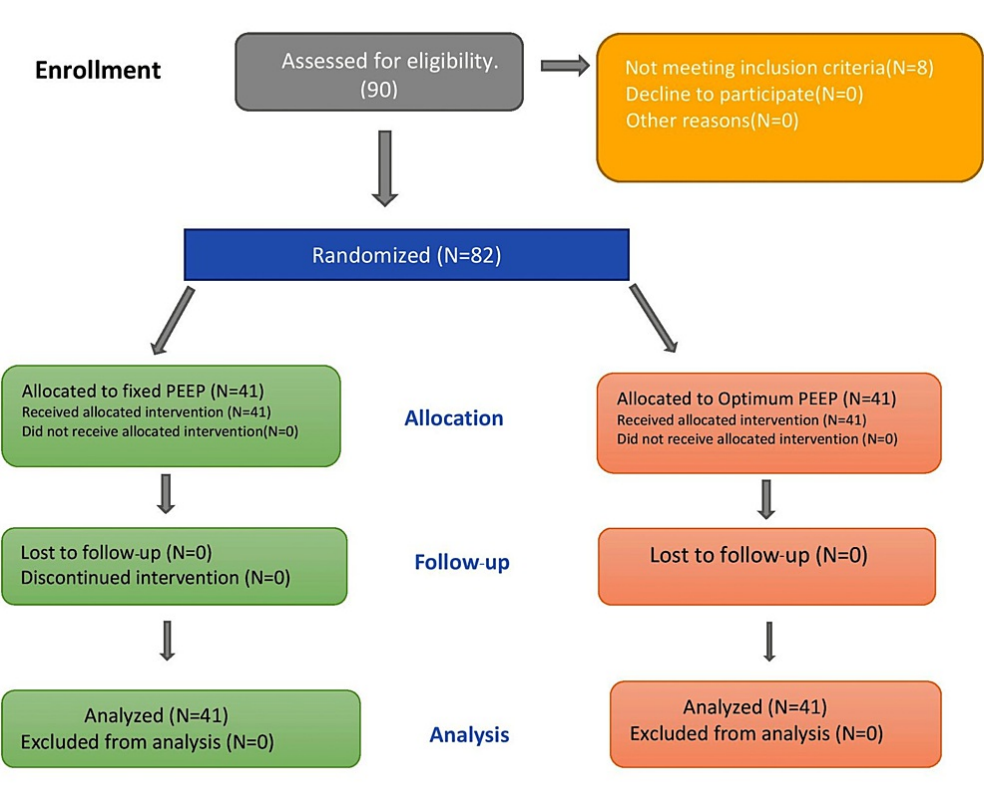
The Consolidated Standards of Reporting Trials (CONSORT) flow diagram.

This study was conducted over one and a half years between March 2020 and August 2021 in AIIMS, New Delhi, a tertiary care center. Ninety patients were screened for inclusion in the study as they presented sequentially for the surgery. Eight patients did not match the inclusion criteria, so they were excluded from the study. All 82 patients who participated in the study were randomized to receive one of the following two interventions using a computer-generated random number list. The Consolidated Standards of Reporting Trials (CONSORT) flow diagram has been provided in Figure 3.

Baseline demographic data are presented in Table 1. All laboratory parameters collected during the preoperative workup of the patient before surgery, like hemoglobin, serum bilirubin, creatinine, and albumin, were comparable between both groups. Further, both groups presented similar American Society of Anesthesiology Physical Status (ASA PS) status and lung ultrasound scores (at time points 1 and 2).

**Table 1:** Baseline demographic variables of patients and lung ultrasound score (LUS). Data are presented as mean±standard deviation (SD), median and interquartile range (IQR), or proportion as applicable. ASA PS: American Society of Anesthesiology Physical Status; PEEP: positive end-expiratory pressure

Parameter	Fixed PEEP (n=41)	Optimum PEEP (n=41)	p-Value
Age (years)	43.1±9.5	38.4±7.3	0.01
BMI	25.6±2.9	25.8±2.24	0.68
Hemoglobin (mg/dL)	11.2±1.9	11.5±1.58	0.806
Serum Bilirubin (mg/dL)	0.4 (0.3-0.45)	0.4 (0.3-0.5)	0.127
Serum Creatinine (mg/dL)	0.6 (0.6-0.7)	0.6 (0.6-0.7)	0.602
Serum Albumin (mg/dL)	4.2 (4-4.4)	4.2 (4.1-4.6)	0.147
ASA PS (I/II)	27/14	31/10	0.234
Lung ultrasound score before intubation (LUS-A)	0 (0,0)	0 (0,0)	0.9705
Lung ultrasound score after intubation (LUS-B)	0 (0-1)	0 (0-1)	0.1752

Median (IQR) use of crystalloids and colloids between Group F and Group O were similar, with the median values being median (IQR) 1.5 (1.5-2) L versus 1.5 (1.5, 1.5) L, p=0.16 and 0 (0,0) L versus 0 (0,0) L, p=0.39, respectively. 

Lung ultrasound score

**Figure 4:**
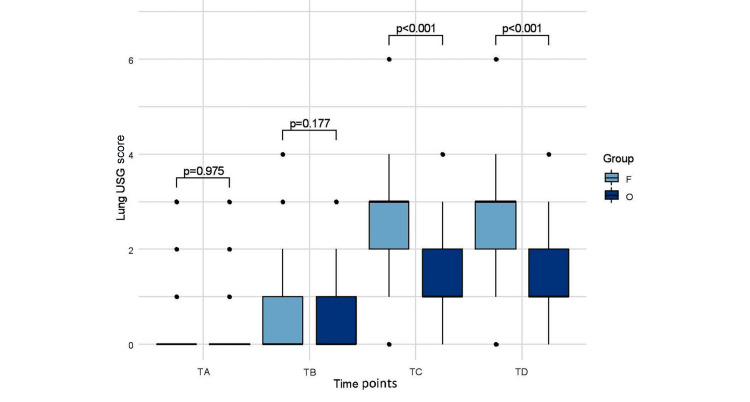
Lung ultrasound score (LUS) in both groups after induction and 30 minutes after extubation. Box-whisker plot showing lung ultrasound aeration score in two groups at different time points (TA=baseline, TB=after induction of anesthesia, TC=before extubation, TD=30 minutes after extubation); p-values were derived from Mann-Whitney U test.

A comparison between the median (IQR) of lung ultrasound scores at different time points in both groups is depicted in Figure 4. Lung USG scores before (LUS-A, p=0.97) and after (LUS-B, p=0.17) induction of anesthesia were similar in both the groups, whereas after deflation of pneumoperitoneum before extubation (LUS-C, p=0.0000) and 30 minutes after extubation (LUS-D, p=0.0000) are significantly higher in fixed PEEP group. Lung USG scores before (LUS-A, p=0.97) and after (LUS-B, p=0.17) induction of anesthesia were similar in both the groups, whereas after deflation of pneumoperitoneum but before extubation (LUS-C, p=0.0001) and 30 minutes after extubation (LUS-D, p=0.0001) are significantly higher in fixed PEEP group. LUSC after deflation of pneumoperitoneum in fixed and optimum PEEP groups were median (IQR) 3 (2-3) versus 1 (1-2), p=0.0001, Mann-Whitney U test and LUS-D 30 minutes after extubation were median (IQR) 3 (2-3) versus 1 (1-2), p=0.0001, Mann-Whitney U test. The lung USG score increased with time from baseline within both groups.

Ventilatory parameters

Ventilatory parameters, including peak airway pressure, plateau pressure, static compliance, and driving pressure at early time points, just after induction (T1), after final positioning but before pneumoperitoneum (T2A), after pneumoperitoneum but before lung recruitment (T2B) were similar between two groups.

Ventilatory parameters between Group F and Group O, after lung recruitment (T3B), were tidal volume (mean 357 mL {SD±35} versus ​362 mL {SD: 22}, p=0.46), PEEP (median, 5 cm H_2_O {IQR: 5-5} versus median 16 cm H_2_O {IQR: 14-18}), peak airway pressure (median 26 cm H_2_O {IQR: 24-28} versus median 30 cm H_2_O {IQR: 28-32} p<0.0001), plateau pressure (median 22 cm H_2_O {IQR: 20-24} versus median 26 cm H_2_O {IQR: 24-28}, p<0.0001), static compliance (32.07±8.36 mL cm H_2_O-1 versus 39.58±8.99 mL cm H_2_O-1, p=0.0002).

Ventilatory parameters between Group F and Group O, after deflation of pneumoperitoneum (T3D), were tidal volume (mean 358 mL {SD: 35} versus 361 mL {SD: 22}; p=0.63), respiratory rate (mean 14.97 {SD: 1.55} versus 15.02 {SD: 1.27}, p=0.877) PEEP (median, 5 cm H_2_O {IQR: 5-5} versus median 8 cm H_2_O {IQR: 6-10}), peak airway pressure (median 19 cm H_2_O {IQR: 18-20} versus median 22 cm H_2_O {IQR: 20-24}, p<0.0001), plateau pressure (median 15 cm H_2_O {IQR: 14-16} versus median 18 cm H_2_O {IQR: 17-20}, p<0.0001), static compliance (37.86±10.29 mL cm H_2_O-1 versus 40.30±8.12 mL cm H_2_O-1, p=0.2267).

**Figure 5:**
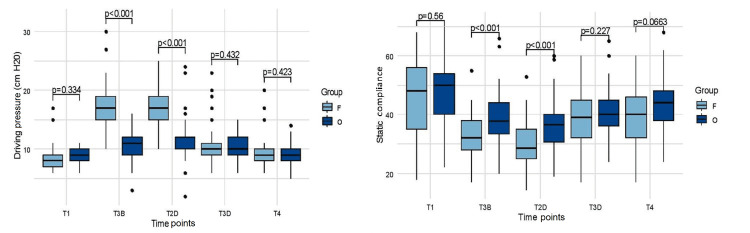
Ventilatory parameter between both groups at different time points. Variation of ventilatory parameter (driving pressure and static compliance) over time (T1=baseline, T3B=after pneumoperitoneum after lung recruitment, T2D=just before deflation of pneumoperitoneum, T3D=after deflation of pneumoperitoneum, T4=30 minutes before extubation); p-values were derived from Mann-Whitney U test.

Ventilatory parameters between Group F and Group O, before extubation (T4), were tidal volume (mean 357.80 mL {SD: 35} versus 362 mL {SD: 22}, p=0.50), respiratory rate (mean 14.90 {SD: 1.48} versus 14.92 {SD: 1.42}, p=1.00) PEEP (median 5 cm H_2_O {IQR: 5-5} versus median 8 cm H_2_O {IQR: 6-10}), peak airway pressure (median 18 cm H_2_O {IQR: 17-19} versus median 22 cm H_2_O {IQR: 19-22}, p=0.0001), plateau pressure (median 14 cm H_2_O {IQR: 13-15} versus median 18 cm H_2_O {IQR: 15-18}, p=0.0002), static compliance (39.10±10.09 mL cm H_2_O-1 versus 42.95±8.35 mL cm H_2_O-1, p=0.0662). Figure 5 compares driving pressure and static compliance at five-time points.

Arterial blood gas

Arterial pH (p=0.06), PaCO_2 _(p=0.59), HCO_3 _(p=0.19, PaO_2_ (p=0.62), P/F ratio (p=0.71), alveolar-arterial gradient (p=0.369), and lactate (p=0.308) were comparable between both groups at all four-time points of ABG analysis.

Correlations

**Figure 6:**
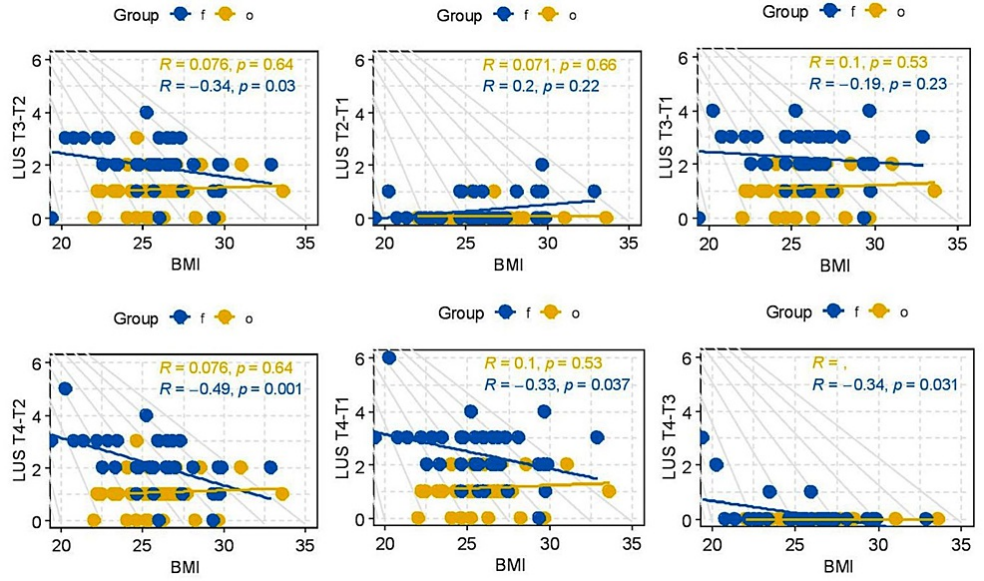
Correlation of lung ultrasound score (LUS) at different points with BMI.

Various study parameters have been correlated against the variation in LUS between different time points for each group. The usage of difference in LUS allows monitoring the evolution of the patient condition during the study and minimizes the dependencies on the baseline condition. Hence the same was used for studying correlations. Figure 6 depicts the correlation between BMI and the difference in LUS at different points for both groups F and O.

It is observed that there is a negative correlation between the difference in LUS score and BMI in fixed-group patients. The difference over time in LUS score is increased in lower BMI patients indicating a more significant worsening of lung aeration in lower BMI patients who received a fixed PEEP intraoperatively. Whereas there is no correlation between BMI and difference in LUS score over time in the optimum PEEP group indicating that optimum PEEP is equally effective in lower BMI as well as higher BMI groups. No significant correlations were observed between the difference in LUS score at different time points and parameters, such as duration of surgery, the difference in P/F ratio, and the difference in A-a gradient.

Hemodynamic data analysis

Two patients in the fixed PEEP group developed hemodynamic changes during the recruitment maneuver, so recruitment was abandoned. The incidence of intraoperative hypotension and bradycardia were similar between the groups as assessed by Fisher's exact test (p=0.181 and p=0.494, respectively, for groups F and O).

Oxygen requirement

**Figure 7:**
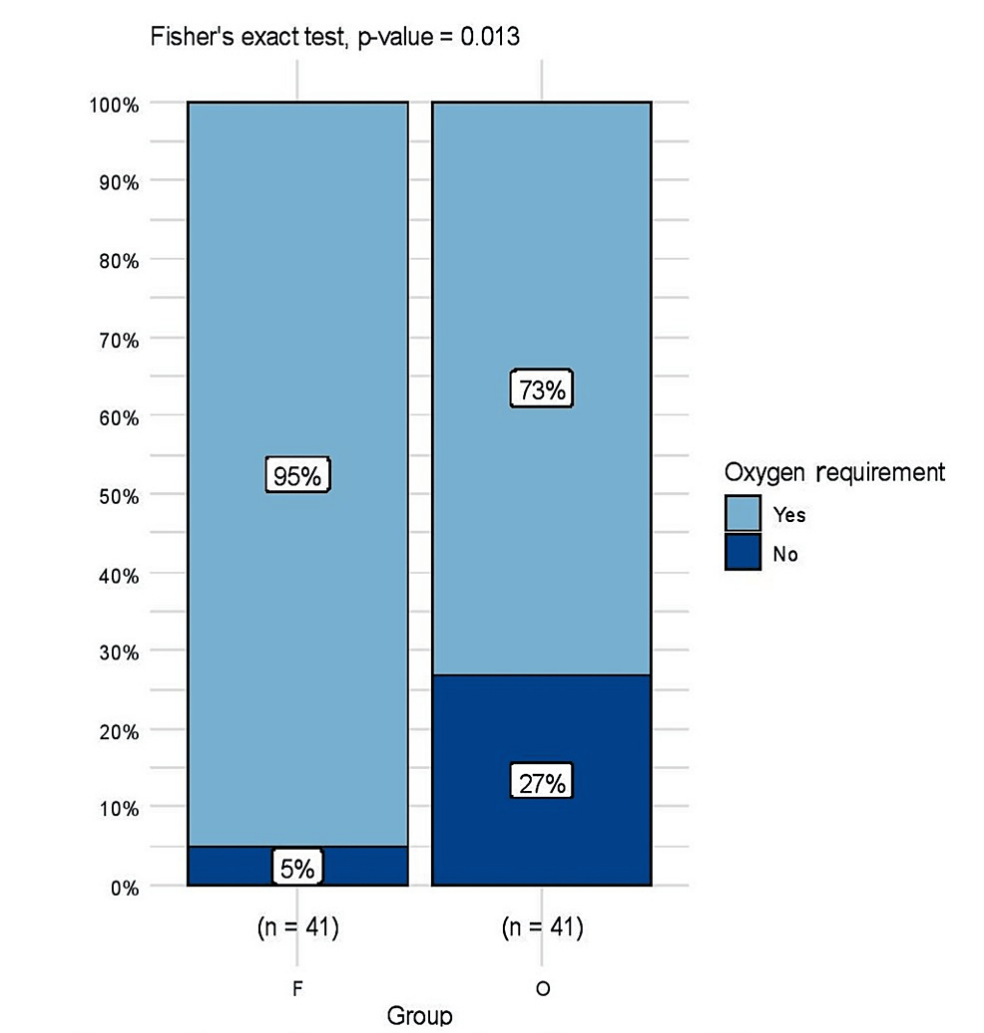
Comparison of number of patients requiring supplemental oxygen for maintaining SaO2>94% in both groups in the postoperative period.

**Figure 8:**
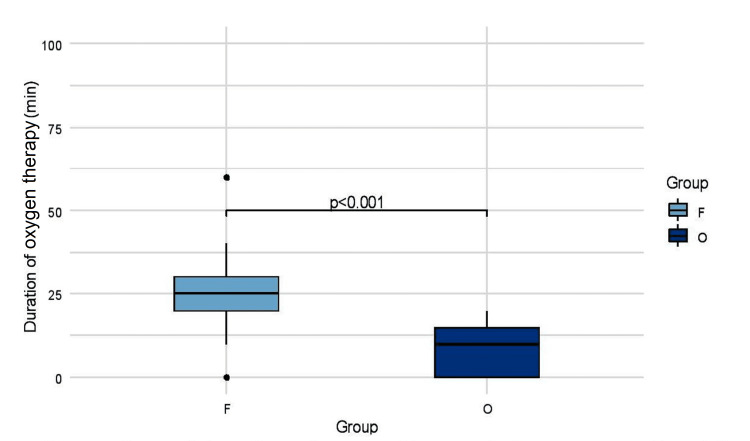
Comparison of duration of oxygen therapy between Group F and Group O.

A comparison between the number of patients requiring postoperative oxygen therapy to maintain SpO_2_ >94% after extubation in PACU between the two groups is shown in Figure 7. There was a statistically significant difference between the two groups - Group F (39 of 41 {95%}} versus Group O (30 of 41 {73%}) (p=0.007, Fisher's exact test). Median (IQR) duration of oxygenation therapy in the first 24 hours of the postoperative period between Group F and Group O differed with statistical significance, with the median (IQR) values being 25 (20-30) minutes versus 10 (0,15) minutes (p=0.0000). Figure 8 compares the duration of oxygen therapy between the two groups. None of the patients required oxygen after being discharged from PACU on their first postoperative day. Also, all the patients were discharged from the hospital on their first postoperative day. We have also noted that no patient required any prolonged hospital stay or required readmission in an emergency or in wards for any surgery or anesthesia-related complication concerning postoperative pulmonary complications within 30 days of discharge.

## Discussion

We in our study, found a significant reduction in the magnitude of postoperative lung atelectasis in adult patients undergoing major laparoscopic gynecological surgery under general anesthesia as assessed by lung ultrasound with the use of compliance-based optimum PEEP as compared with fixed PEEP. Optimum PEEP also provided lower intraoperative driving pressures, better postoperative oxygenation, reduced requirement of postoperative supplemental oxygen, and a possible decrease in POPC without any significant hemodynamic alterations. Généreux et al. reported better lung aeration scores, higher respiratory compliances, and better gas exchange using recruitment maneuver (RM) with PEEP compared to zero PEEP in open gynecological surgery [11]. Our study adds that an optimum PEEP based on lung compliance reduces atelectasis over a fixed PEEP.

Östberg et al. in 2018 reported that PEEP alone without recruitment maneuver was found to be effective in reducing general anesthesia-induced atelectasis in patients undergoing non-abdominal surgery [15]. Deng et al., in their meta-analyses of several randomized controlled trials (RCTs), found that low tidal volume ventilation with moderate (5-8 cm H_2_O) to high PEEP (>9 cm H_2_O) reduced the risk of postoperative pulmonary complications and that moderate to high PEEP with recruitment maneuver specifically reduced the risk of atelectasis in surgical patients [16]. Our study results also comply with the above results. We found a statistically significant lower driving pressure in the optimum PEEP group compared to the fixed PEEP group during pneumoperitoneum. Past studies also consistently correlate low driving pressure with better lung aeration and less lung atelectasis [16,17]. Lower driving may be the reason for a better-modified lung ultrasound score in the optimum PEEP group compared to the fixed PEEP group. Better modified lung ultrasound score may be why fewer patients require oxygen supplementation to maintain oxygen saturation of more than 94% in PACU and the first postoperative day in the optimum PEEP group compared to the fixed PEEP group.

Zhang et al. in 2021 evaluated the effect of driving pressure-guided PEEP versus fixed PEEP of 6 cm H_2_O in open abdominal surgery on the incidence of clinically significant POPC on the seventh postoperative day. They found a median PEEP of 10 cm H_2_0 in the individualized group. The incidence of clinically significant POPC and driving pressure was significantly lower in the individualized group than in the fixed group [17].

An individual patient data meta-analysis found that a higher driving pressure during general anesthesia is one of the most critical ventilatory parameters associated with a higher incidence of postoperative pulmonary complications. A lower intraoperative driving pressure has been associated with reduced postoperative pulmonary complications. In our study, by implementing compliance-based PEEP, we have achieved the same indirectly and found that appreciation of the individualized setting of PEEP is beneficial over fixed PEEP in anesthetized patients.

From a physiological perspective, compliance-based PEEP that also delivers the lowest driving pressure avoids both over-distension and atelectasis [18-20]. It is well known that there is a significant inter-individual variation in respiratory system compliance during surgery [11], and PEEP titration during surgery reduces postoperative atelectasis [21]. We have also found in our study that PEEP titration following a recruitment maneuver was not associated with significant hemodynamic instability in an optimized ASA 1/2 patient.

Our study is different from previous RCTs such that we have kept individualized titrated PEEP based on lung compliance throughout the intraoperative period in the study group which was titrated again during surgery at one-hour intervals during pneumoperitoneum and again after deflation of pneumoperitoneum considering the variation in respiratory system compliance with surgical time points. During laparoscopic pelvic surgery, it is not only the general anesthesia that contributes to atelectasis but also the Trendelenburg position, the raised intraabdominal pressure by the creation of pneumoperitoneum, and the duration of surgery and anesthesia all contribute to atelectasis. Our PEEP titration strategy considered both individualized respiratory system compliance concepts and changes in compliance over time during the intraoperative period.

Valenza et al., in a study, found that a combination of beach chair position and PEEP improved oxygenation during pneumoperitoneum induction in morbidly obese patients [11]. In our study, we found a more significant worsening of lung aeration in lower BMI patients who received a fixed PEEP intraoperatively. Optimum PEEP based on individualized lung compliance was found equally effective in lower BMI and higher BMI groups in preventing lung atelectasis.

In the PROVHILO trial, there was no significant difference in POPC between the high and low PEEP groups, however, they reported a high incidence of hemodynamic instability and vasopressor requirement in the high PEEP group [2]. Similarly, another RCT by PROBESE collaborative group shows an insignificant effect of high PEEP with recruitment maneuver in obese patients [21]. However, they reported better postoperative oxygenation in the high PEEP group. The lack of benefit from the high PEEP strategy in these two trials is likely due to the failure to consider an individualized PEEP titration strategy. Though we have found a possible reduction in postoperative lung atelectasis as assessed by lung ultrasound with optimum PEEP, it needs further validation in large randomized controlled trials.

Strength of study

Our study is strengthened by the fact that there is a change in lung mechanics during the intraoperative period. To combat the change in lung mechanics during the intraoperative period, we did frequent lung recruitment maneuvers followed by applying optimum PEEP in the study group. This study highlights the actual effect of optimum PEEP on lung ultrasound scores. The patient population is homogeneous regarding procedure and position. So our finding can be generalized in laparoscopic surgery in supine and Trendelenburg positions.

Limitations

This study is limited by sample size and narrow inclusion criteria. We included only laparoscopic gynecological surgery with average body weight, so it can not be generalized to laparoscopic surgery in obese or overweight patients. Upper abdominal laparoscopic surgery will have more effect on lung compliance, and was also not included. Although lung ultrasound is a validated tool for diagnosing lung atelectasis, it is operated-dependent and not the gold standard. We did frequent recruitment maneuvers in both groups at a one-hour time interval; the time interval chosen was arbitrary. Though the amount of intraoperative fluid administered was similar in both groups, fluid therapy was not "protocolized" in our study.

## Conclusions

Compliance-based intraoperative PEEP titration reduced postoperative lung atelectasis as assessed by lung ultrasound in adult patients undergoing major laparoscopic gynecological surgery. We have also found that there is improved oxygenation in these patients and hence a decreased requirement of postoperative supplemental oxygen and, whenever needed, a lesser duration of supplemental oxygen as compared with fixed group patients; but these findings need to be further verified in larger randomized controlled trials.
